# LVAD specific infection with *Coxiella burnetii* treated with heart transplantation and antibiotic suppression

**DOI:** 10.1007/s10096-026-05464-x

**Published:** 2026-03-16

**Authors:** Nikolaos Cholevas, Lucka Kuntner, Evgenij Potapov, Jan Knierim, Andrej Trampuz, Annette Moter, Judith Kikhney, Felix Schoenrath

**Affiliations:** 1 German Heart Center of the Charité, Berlin, Germany; 2 Charité - University Medicine Berlin, Berlin, Germany

**Keywords:** LVAD infection, Coxiella burnetii, Heart transplantation

## Abstract

We report a rare case of LVAD infection caused by *Coxiella burnetii* in a 43-year-old male. The infection was diagnosed after CT-guided abscess sampling, FISHseq analysis, and serology. Conservative therapy with doxycycline and hydroxychloroquine proved unsuccessful. After LVAD removal and heart transplantation, the patient has remained infection-free for three years under antibiotic suppression.

## Introduction

Infections are a serious complication of ventricular assist device (VAD) therapy and are associated with substantial morbidity and mortality [1]. As removing or exchanging the device is often not feasible, long-term antibiotic treatment of 6–12 weeks or longer is commonly necessary. We report a clinical case of a systemic Left Ventricular Assist Device (LVAD) specific infection with *Coxiella burnetii*, a microorganism rarely associated with VAD infections [2].

## Case report

A 43-year-old male, with history of an LVAD implantation 11 years earlier due to end-stage ischemic heart failure, presented with a CT-detected abscess around the LVAD during routine assessment for the maintenance of the heart transplantation listing status.

In his past history, the patient had a chronic driveline infection 7 years prior to the current presentation with isolation of Corynebacterium amycolatum in driveline swab. The Positron Emission Tomography-Computed Tomography (PET-CT) scan revealed at that time a localized driveline infection with no involvement of the central parts of the LVAD. After a suppression therapy with doxycycline 100 mg q24h for a total duration of 12 months the infection was resolved. After the cessation of suppression therapy, no clinical or microbiological recurrence of the driveline infection occurred.

**Fig. 1:**
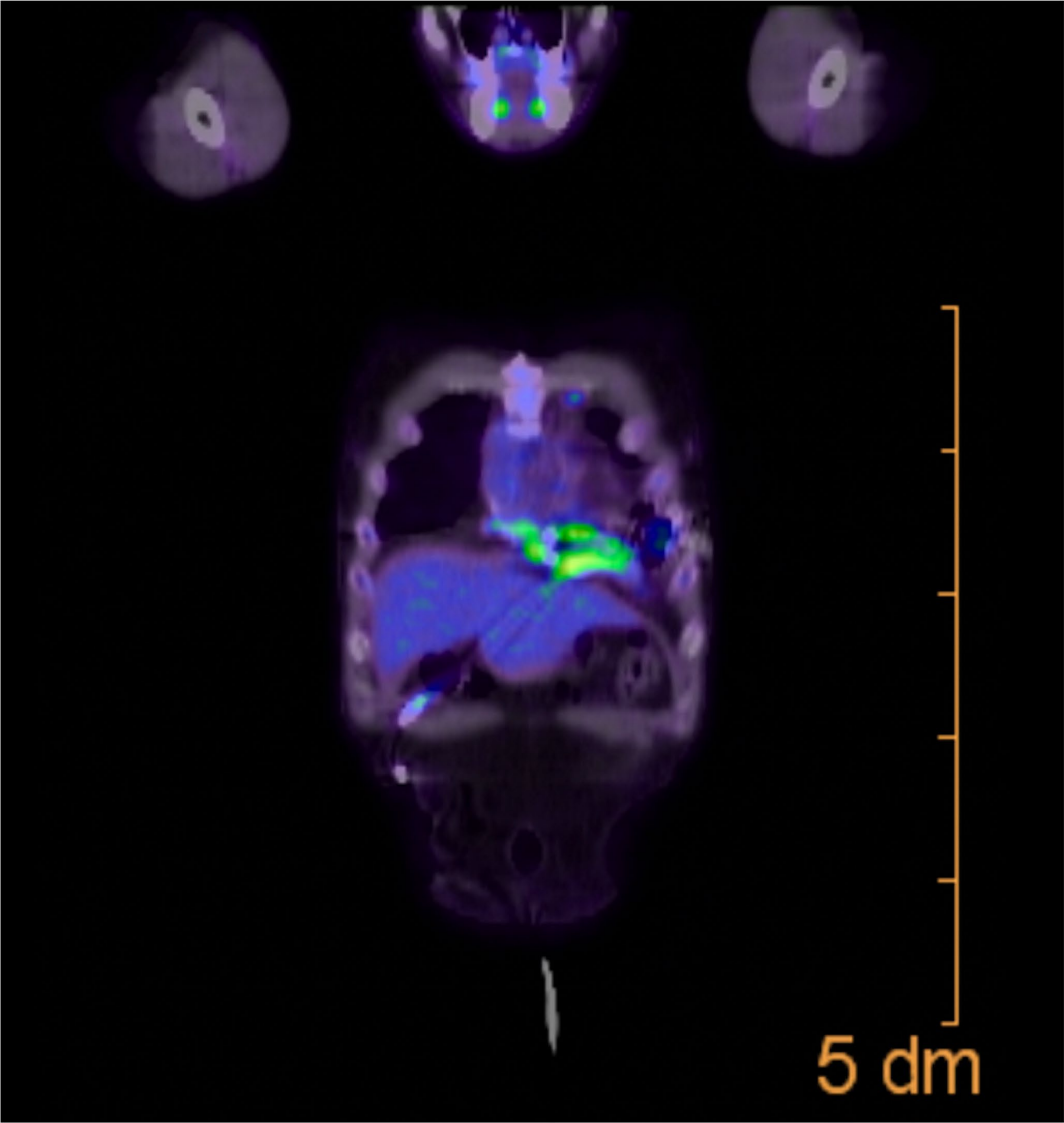
PET-CT scan indicating a multifocal endoplastitis of the LVAD including a hypermetabolic abscess in the upper mediastinum

At the current presentation, the patient had no symptoms or clinical signs of driveline or systemic infection and the inflammation markers were normal. The transesophageal echocardiogram (TOE), blood cultures, and driveline swab yielded negative results. The subsequent PET-CT revealed extensive multifocal endoplastitis affecting the entire LVAD driveline, LVAD aggregate (pump housing), and outflow graft junction. Additionally, a hypermetabolic abscess in the upper mediastinum in contact with the aorta and truncus pulmonalis was detected (Fig. 1).

**Fig. 2:**
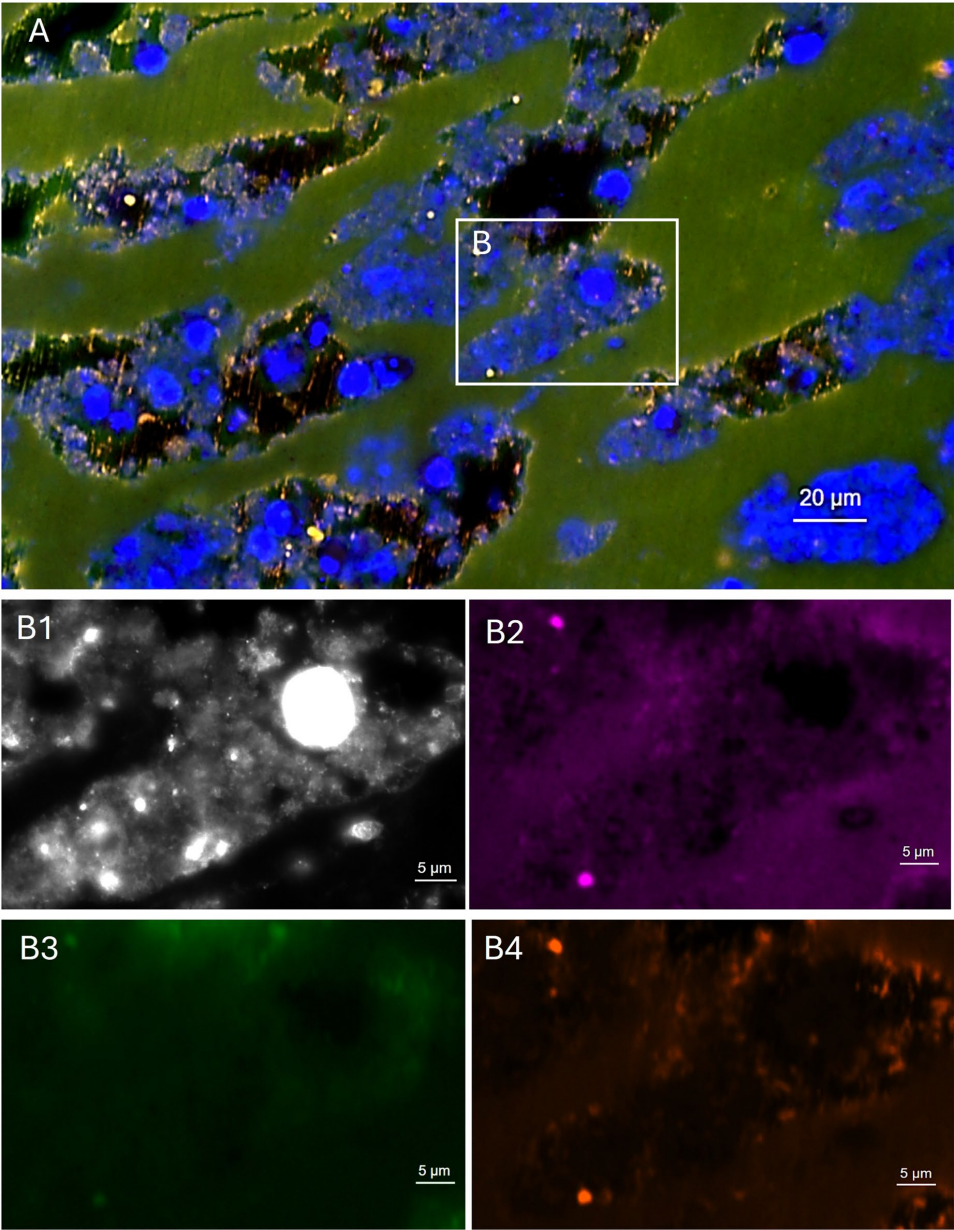
FISH of the abscess aspirate shows tissue fragments, in which the nucleic acid stain DAPI (blue) reveals intracellular pleomorphic structures next to the host cell nuclei that are in line with a *Coxiella burnetii* infection (A, overview and B1, higher resolution of DAPI stain displayed in black/white for better contrast). Only very few bacteria are detected by the pan-bacterial FISH probe (magenta, B2), the *Coxiella*-specific probe Coxb0187 (orange, B4), without autofluorescence signals or the unspecific binding of control FISH probe NON-EUB338 (both green, tissue background and non-sense FISH probe)

A diagnostic CT-guided sampling of the abscess around the outflow graft revealed an infection with *C. burnetii* through Fluorescence In Situ Hybridization combined with 16 S rRNA-gene polymerase chain reaction and sequencing (FISHseq) (16 S-rRNA gene amplification and sequencing with 100% identity over 501 base pairs) (Fig. 2) [3]. When inquired about potential animal contact, the patient disclosed a friendship with a shepherd in his hometown. The patient had no symptoms of systemic infection and no specific manifestation of chronic Q Fever such as splenomegaly, rash or kidney involvement. The serology results for *C. burnetii* were positive (ELISA phase II IgG > 500 U/ml, ELISA phase II IgM positive, IFA phase I IgG titer 1:525288, IFA phase II IgG titer 1:2097152).

A treatment with doxycycline 100 mg q12h and hydroxychloroquine 200 mg q8h was initiated. After five months of combined treatment, a follow-up CT demonstrated a worsening LVAD-associated infection with a slight increase in abscess size. Consequently, the listing status was upgraded to High Urgency (HU), leading to the patient undergoing heart transplantation two months later. Post-surgery, due to hydroxychloroquine intolerance (nausea and vomiting), the oral therapy was adapted to ciprofloxacin 250 mg q12h and doxycycline 100 mg q12h and a long-term therapy after the heart transplantation was recommended. During a 3-year follow-up, no clinical, laboratory, echocardiographic, or radiological recurrence of the disease was observed. The serology results were continuously improving (3 years after heart transplantation: phase I IgG titer 1:256, ELISA phase II IgG 46.8 U/ml and ELISA phase II IgM negative).

Long-term suppression antibiotic therapy was decided due to the immunosuppression required after the heart transplantation.

**Fig. 3:**
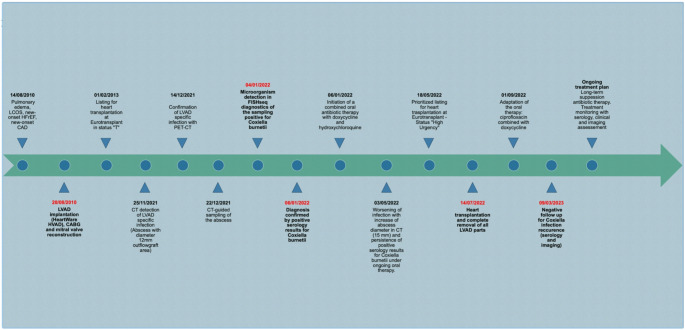
Timeline presenting the disease course starting from the initial heart failure diagnosis LVAD implantation and ending with the heart transplantation and the ongoing successful treatment of the LVAD specific *Coxiella burnetii* infection. LCOS: Low Cardiac Output Syndrome, HFrHF: Heart Failure with reduced Ejection Fraction, CAD: Coronary Artery Disease, LVAD: Left Ventricular Assist Device, CABG: Coronary Artery Bypass Grafting, PET-CT: Positron Emission Tomography-Computed Tomography, CT: Computed Tomography, FISHseq: Fluorescence In Situ Hybridization combined with 16 S rRNA-gene polymerase chain reaction and sequencing, “T”: transplantable

The timeline of the 14-year disease course from the initial heart failure diagnosis and LVAD implantation to the heart transplantation and the so far successful treatment of the LVAD specific *C. burnetii* infection is illustrated in Fig. 3.

## Discussion

To our knowledge, to date, only one case of systemic LVAD-specific infection with *C. burnetii* has been published [2]. While *C. burnetii* is well-established as a cause of culture-negative endocarditis and infections of cardiac implantable electronic devices (CIEDs) [4, 5], for which doxycycline and hydroxychloroquine treatment spanning 12–18 months is recommended [6], no specific treatment guidelines currently address VAD-specific infections involving this microorganism.

In our case, the conservative approach employing doxycycline and hydroxychloroquine proved ineffective, as evidenced by progression of the abscess size on follow-up CT imaging. A potential explanation may be the formation of biofilms on the LVAD surface, similar to those seen in endocarditis [7], which are recalcitrant to eradication by antibiotic therapy alone.

Following complete device removal, heart transplantation, and subsequent combined antimicrobial therapy, the patient demonstrated a sustained clinical and microbiological response, with no evidence of recurrent infection during a three-year follow-up. This favorable outcome was supported not only by the absence of clinical or imaging signs of relapse, but also by a significant and persistent improvement in serological markers.

It is important to acknowledge that lifelong immunosuppression represents a major challenge in the long-term management of chronic Coxiella burnetii infection. In our case, three years after heart transplantation, the combination of complete removal of the infected LVAD and prolonged oral antimicrobial therapy appears to have mitigated the risk of recurrence so far, despite the patient’s immunosuppressed status. Decisions regarding the duration of antimicrobial treatment should be individualized and guided primarily by serial serological monitoring—particularly phase I IgG titers—along with careful clinical and imaging evaluation when indicated.

We therefore conclude that:

1. The diagnosis of an LVAD-specific infection with *C. burnetii* poses significant diagnostic and therapeutic challenges.
2. In this case, antimicrobial therapy alone was insufficient to control the infection.
3. The combination of complete device removal and prolonged antibiotic treatment demonstrated sustained infection control, despite ongoing immunosuppression following heart transplantation.

In light of this case, we suggest screening for atypical bacteria in patients with confirmed VAD-specific infections and an unclear infection pathogenesis. In these cases, FISHseq analysis can be helpful in the diagnostic process [8, 9]. This proactive approach may enhance diagnostic precision and guide more effective therapeutic strategies.

## Data Availability

No datasets were generated or analysed during the current study.
